# Patient challenges across the implant-based breast reconstruction journey: A qualitative descriptive study

**DOI:** 10.1016/j.apjon.2026.101046

**Published:** 2026-10-15

**Authors:** Xin Zhang, Jiaqin Guan, Jing Li, Jiajia Qiu, Liqin Chen, Zhenqi Lu, Xiaoju Zhang

**Affiliations:** a Department of Nursing, Shanghai Cancer Center, Fudan University, Shanghai, China; b Department of Oncology, Shanghai Medical College, Fudan University, Shanghai, China

**Keywords:** Breast neoplasms, Mammaplasty, Breast implants, Qualitative research

## Abstract

**Objective:**

To explore patient-perceived challenges across different phases of the implant-based breast reconstruction journey following breast cancer surgery, and to inform stage-specific supportive nursing care.

**Methods:**

A qualitative descriptive design was used. Eighteen women who had undergone implant-based breast reconstruction at a cancer hospital in Shanghai, China, were recruited through purposive sampling to achieve variation in age, education, and treatment experience. Semi-structured interviews were conducted between January and March 2025. Timing Theory informed the temporal structure of the interview guide and the temporal organization and interpretation of findings. Data were analyzed using thematic analysis, with codes and themes developed inductively from participants’ narratives without using Timing Theory as a coding framework. Trustworthiness was enhanced through independent coding, team discussions, participant feedback, and detailed documentation of the analytic process.

**Results:**

Four data-derived themes were identified. These themes reflected challenges across different phases of the reconstruction journey: (1) Uncertainty regarding adverse outcomes of implant-based reconstruction; (2) Conflicting demands of functional recovery and implant shaping; (3) Conflicting care guidance during radiotherapy; and (4) Psychological burden of living with a permanent implant.

**Conclusions:**

Women undergoing implant-based breast reconstruction experience distinct challenges that evolve throughout the reconstruction journey. Based on these findings, nursing strategies addressing these challenges, including clearer decision-related information, rehabilitation guidance, consistent treatment-related communication, and psychosocial support, may help patients navigate reconstruction-related difficulties. Addressing these challenges may improve adaptation, strengthen self-management, and enhance the overall reconstruction experience.

## Introduction

Breast cancer remains the most commonly diagnosed cancer among women worldwide and a major public health concern. According to the latest Global Cancer Statistics 2024, approximately 2.4 million women were newly diagnosed with breast cancer and 694,000 died from the disease worldwide in 2024.^1^ In China, there were approximately 357,000 new cases of female breast cancer in 2022.^2^ Fortunately, because of better screening and treatments, more women are surviving the disease.^1^ As a result, many patients now pay more attention to their quality of life after surgery. Breast reconstruction has therefore become an important option for women undergoing breast cancer surgery and is often performed during the same procedure as the cancer surgery.^3^, ^4^

Implant-based reconstruction is a popular choice because it is less invasive, has a faster recovery, and provides stable results.^5^ However, most studies have focused on surgical techniques, safety, and complications.^6^, ^7^ Less is known about the patient challenges associated with implant-based breast reconstruction, including how patients manage daily care or cope with emotional concerns.^8^ Most research uses surveys to measure satisfaction or complication rates, which cannot fully capture a patient's personal experience or their worries.^9^ In particular, most qualitative studies on implant-based reconstruction have focused on how patients make their decisions.^10^, ^11^ These studies have provided valuable insights into decision-making processes, but they offer limited understanding of patients' experiences after reconstruction. Nevertheless, the existing literature provides insufficient understanding of patient-perceived challenges throughout the reconstruction process. More importantly, existing research rarely examines how these challenges vary across different stages of treatment or how they are shaped by the specific characteristics of implant-based reconstruction.^12^ For example, more knowledge is needed about how patients manage their implants in daily life, the difficulties they face when wearing shaping garments, and how they coordinate their care across different stages of treatment.

Using a theory-guided approach can facilitate understanding of how patients’ concerns and challenges evolve throughout the reconstruction trajectory. Timing Theory,^13^ developed by Cameron and Gignac, was originally proposed to explain how support needs change over time among family caregivers of stroke survivors. The theory emphasizes that individuals experience distinct challenges, priorities, and support needs at different stages of a health-related journey. Although originally developed in the context of stroke caregiving, its temporal perspective has been increasingly applied to health conditions characterized by dynamic care needs over time.

Implant-based breast reconstruction is not a single surgical event but a prolonged treatment journey that includes decision-making, postoperative recovery, adjuvant treatment, and long-term adaptation. Patients may encounter different concerns at each phase. Therefore, Timing Theory provided a useful conceptual framework for understanding how patient challenges evolve across the reconstruction trajectory. Using Timing Theory as a sensitizing conceptual framework, the present study aimed to identify patient-perceived challenges across the distinct phases of implant-based breast reconstruction (decision-making, postoperative recovery, adjuvant treatment, and long-term adaptation). The theory guided attention to temporal variation in experience and informed the structure of data collection and the presentation of findings, while theme development remained inductive. Implications for patient-centered nursing care were derived from these findings.

## Methods

### Design and participants

A qualitative descriptive design was used because it provides a comprehensive summary of participants' experiences in everyday terms.^14^ Timing Theory^13^ subsequently served as a sensitizing conceptual framework to inform the development of the interview guide and to support the subsequent organization and interpretation of the inductively derived findings. Between January and March 2025, 18 women who had implant-based breast reconstruction at the Shanghai Cancer Center, Fudan University were recruited. This study followed the COREQ checklist to ensure clear and complete reporting.^15^

Purposive sampling was used to select participants. Eligible patients were identified and approached during their follow-up appointments or while receiving comprehensive breast cancer treatment. Recruitment was conducted by a research nurse who was not involved in the participants' clinical care. To ensure a diverse sample, participants were selected to represent a range of characteristics, including different ages, education levels, surgery types, and follow-up treatments. The inclusion criteria were (Ⅰ) aged 18 years or older with a confirmed diagnosis of breast cancer (Ⅱ) had undergone implant-based breast reconstruction and were in a stable condition (Ⅲ) able to communicate clearly and express their experiences (Ⅳ) willing to participate and provided informed consent. The exclusion criteria were (Ⅰ) end-stage disease or critical condition (Ⅱ) diagnosed mental disorders (Ⅲ) risk of self-harm (Ⅳ) language or communication difficulties. The final sample size was determined by data saturation. After 16 interviews, no new ideas or themes emerged. Two more interviews were then conducted to confirm saturation, resulting in a total of 18 participants. All participants understood the study and signed informed consent before participation.

### Data collection

Data were collected by the first author from January to March 2025 through face-to-face, semi-structured interviews. The interviews were conducted in a private ward conference room. Before the interviews, the researcher explained the purpose of the study. Participants were told that participation was voluntary and that their information would be kept confidential. With permission, the interviews were audio-recorded and lasted 20–40 minutes, with an average duration of approximately 32 minutes. During the interviews, the researcher took notes on non-verbal cues such as facial expressions. She also used follow-up questions to clarify key points. Leading questions were avoided to ensure the accuracy of the data.

The interview guide was developed through a review of the literature, discussions within the research team, and Timing Theory as a sensitizing framework. Timing Theory informed the temporal structure of the questions, prompting participants to reflect on challenges and care experiences during preoperative decision-making, postoperative recovery, adjuvant treatment, and long-term adaptation. It did not prescribe specific codes or themes. The process followed Kallio's five-step method,^16^ which included (I) defining the need for a semi-structured interview (II) reviewing existing knowledge (III) drafting the initial interview guide (IV) pilot testing the guide with a small sample; and (V) finalizing the interview guide. Prior to formal data collection, two pilot interviews were conducted with a separate group of two eligible women who had undergone implant-based breast reconstruction. These pilot participants were chosen based on their availability and willingness to provide feedback on the interview guide. The pilot participants reported that the questions were understandable and relevant to their experiences. No major issues were identified, and no substantive changes were made to the interview guide based on their feedback. Data from the pilot interviews were not included in the final analysis. Based on the pilot results and team discussions, the interview guide was revised and finalized. The main interview questions were (I) How did you experience and manage implant-related care across different stages of the reconstruction journey, including preoperative decision-making, postoperative recovery, adjuvant treatment, and long-term adaptation (II) What instructions on implant-related care did you receive at different stages, and how did you apply them in your daily care (III) What difficulties did you experience in implant-related nursing care, and what unmet needs or suggestions do you have (IV) What were your feelings and experiences when performing implant-related care at different stages?

### Data analysis

All interviews were transcribed verbatim within 24 hours. Each participant was assigned a code (P1–P18), and identifying information was removed to protect confidentiality. The data were analyzed using inductive thematic analysis following Braun and Clarke's six-phase approach.^17^ Initial codes were generated from participants' narratives. Two researchers independently familiarized themselves with the transcripts and generated initial codes, after which they compared their interpretations and discussed emerging patterns. Through iterative discussion, codes were refined and grouped into candidate themes and subthemes, which were reviewed and further refined against the dataset. Differences in interpretation were discussed throughout the analytic process to deepen interpretation and refine the developing themes. Timing Theory was not used as a coding framework or to generate themes. Instead, it informed data collection and was subsequently used to organize and interpret the inductively derived findings according to their temporal context within the reconstruction journey. The findings were situated within four relevant phases—preoperative decision-making, postoperative recovery, adjuvant treatment, and long-term adaptation—to describe their temporal context and facilitate presentation. Challenges that extended across more than one phase were considered cross-phase issues rather than being assigned exclusively to a single phase.

### Trustworthiness of data

To ensure the quality and trustworthiness of the study, several strategies were employed. Data collection and analysis were conducted concurrently, and participant recruitment continued until no new themes emerged. Two researchers independently reviewed the transcripts and developed initial codes, and their interpretations were discussed throughout the analytic process to deepen interpretation and refine the developing themes. The interviewer was a master's nursing student with clinical experience in breast cancer care, and the analysts had professional backgrounds in oncology nursing. Although the researchers were familiar with the clinical context of implant-based breast reconstruction, none had a direct clinical care relationship with the participants. The research team acknowledged that these professional backgrounds might influence data interpretation and therefore engaged in regular team discussions to reflect on coding decisions and theme development. Detailed records of coding decisions, theme development, and analytical discussions were maintained throughout the study to support transparency in the analytic process. All authors reviewed and refined the final categories and themes to ensure that interpretations remained grounded in the data. In addition, detailed descriptions of participant characteristics, sampling procedures, and study context were provided to facilitate understanding of the findings. Furthermore, to enhance confirmability and credibility, the preliminary findings (rather than full interview transcripts) were shared with two participants for member checking, and both confirmed that the interpretations resonated with their experiences.

## Results

### Participant characteristics

Among the 18 participants, 17 (94.4%) were married and one (5.6%) was single. Nine participants (50.0%) had a bachelor's degree or higher, eight (44.4%) had college-level education, and one (5.6%) had completed high school. Reconstruction types included direct-to-implant reconstruction, two-stage implant reconstruction, and implant reconstruction with autologous tissue. Two participants (11.1%) had received neoadjuvant therapy. Most surgeries were performed between 2023 and 2025. Four participants (22.2%) reported postoperative complications, including infection (*n* = 2), seroma (*n* = 1), and capsular contracture (*n* = 1). Participant characteristics are presented in Table 1.

**Table 1:** General characteristics of participants (*N* = 18).

ID	Age group (years)	Education	Reconstruction type	Time since surgery	Postoperative complications	Adjuvant therapy	Neoadjuvant therapy
P1	40–59	College	Direct-to-implant reconstruction	6–12 months	None	Chemotherapy, targeted therapy	No
P2	40–59	College	Two-stage implant reconstruction	1–3 years	None	Chemotherapy, radiotherapy, targeted therapy	No
P3	18–39	Bachelor	Two-stage implant reconstruction	6–12 months	Infection	Chemotherapy, targeted therapy, immunotherapy	No
P4	18–39	Bachelor	Direct-to-implant reconstruction	> 3 years	Seroma	Chemotherapy, radiotherapy, targeted therapy, immunotherapy	No
P5	40–59	Bachelor	Two-stage implant reconstruction	6–12 months	None	Chemotherapy, targeted therapy	No
P6	40–59	College	Direct-to-implant reconstruction	6–12 months	None	Chemotherapy, radiotherapy, targeted therapy, endocrine therapy	No
P7	18–39	College	Two-stage implant reconstruction	< 6 months	Infection	Chemotherapy, targeted therapy	No
P8	40–59	College	Two-stage implant reconstruction	< 6 months	None	Chemotherapy, radiotherapy, endocrine therapy	No
P9	18–39	College	Two-stage implant reconstruction	1–3 years	None	Chemotherapy, targeted therapy, radiotherapy	Yes
P10	18–39	College	Direct-to-implant reconstruction	6–12 months	None	Radiotherapy	No
P11	40–59	Bachelor	Implant reconstruction with autologous tissue	> 3 years	None	Chemotherapy, targeted therapy	No
P12	40–59	Bachelor	Direct-to-implant reconstruction	< 6 months	None	Chemotherapy, targeted therapy	No
P13	18–39	Bachelor	Two-stage implant reconstruction	1–3 years	None	Chemotherapy, radiotherapy, targeted therapy	No
P14	18–39	Bachelor	Two-stage implant reconstruction	6–12 months	None	Chemotherapy, radiotherapy, targeted therapy	No
P15	≥ 60	High school	Direct-to-implant reconstruction	1–3 years	Capsular contracture	Chemotherapy	No
P16	18–39	Bachelor	Implant reconstruction with autologous tissue	6–12 months	None	Targeted therapy, immunotherapy, radiotherapy	Yes
P17	18–39	Master	Two-stage implant reconstruction	< 6 months	None	Not started	No
P18	40–59	College	Two-stage implant reconstruction	6–12 months	None	Chemotherapy, radiotherapy, targeted therapy	No

### Interview results

Four data-derived themes were identified through inductive thematic analysis: Uncertainty Regarding Adverse Outcomes of Implant-Based Reconstruction; Conflicting Demands of Functional Recovery and Implant Shaping; Conflicting Care Guidance During Radiotherapy; and Psychological Burden of Living With a Permanent Implant. Timing Theory was subsequently used to situate these findings within the temporal context of the reconstruction journey. Some challenges extended across more than one phase and were therefore considered cross-phase issues. A summary of the themes and subthemes is shown in Table 2.

**Table 2:** Themes, subthemes and example codes.

Theme	Subtheme	Example codes
Uncertainty regarding adverse outcomes of implant-based reconstruction	Uncertainty about the impact of radiotherapy on implant outcomes	Worried that radiotherapy might deform the implant; concerned that reconstruction results might worsen after radiotherapy
	Uncertainty about the risk of future revision surgery	Worried that the implant might need replacement; uncertain whether another operation would be required
Conflicting demands of functional recovery and implant shaping	Concerns about implant displacement limiting physical activity	Afraid to raise the arm too high; avoided carrying heavy objects; worried that excessive movement might affect implant stability
	Discomfort related to wearing shaping garments	Felt pain or pressure when wearing the shaping bra; felt tightness and discomfort during prolonged wear; stopped wearing the shaping bra because of discomfort
	Limited awareness of early implant-related complications	Did not recognize early signs of infection; thought redness was normal healing; discharge education focused only on wound infection
Conflicting care guidance during radiotherapy	Conflicting recommendations on shaping garment use during radiotherapy	Received different advice from different doctors; unsure whether to continue wearing the shaping bra during radiotherapy; felt confused by inconsistent instructions
Cross-phase challenge∗	Difficulty recognizing delayed implant-related complications	Unable to distinguish treatment effects from implant problems; had never heard of capsular contracture before; hardness noticed during/after adjuvant therapy
Psychological burden of living with a permanent implant	Persistent sense of foreignness	Felt the reconstructed breast was not truly their own; could clearly feel the implant when touched; found it difficult to fully adapt
	Anxiety triggered by informal information	Became worried after reading negative information online; felt anxious after hearing other patients' experiences

∗ The cross-phase challenge of recognizing delayed implant-related complications spans multiple phases of the reconstruction journey and does not constitute a fifth theme.

#### Theme 1. Uncertainty regarding adverse outcomes of implant-based reconstruction

During the preoperative phase, patients expressed considerable uncertainty regarding potential adverse outcomes of implant-based reconstruction. Two interrelated concerns emerged: the impact of radiotherapy on implant outcomes, and the risk of future revision surgery. These uncertainties often stemmed from insufficient preoperative information and remained unresolved at the time of decision-making.

##### Uncertainty about the impact of radiotherapy on implant outcomes

Some patients worried about the effects of radiotherapy on implant integrity and long-term appearance. Preoperative discussions often did not provide patients with a clear understanding of how radiotherapy might influence implant outcomes, leading them to rely heavily on physicians' recommendations. When reflecting on their decisions, some patients reported that they had not fully considered the possible interactions between radiotherapy and implants. *As P16 described, “I didn't understand anything about implant lifespan or the effects of radiotherapy. The doctor said it was okay to put in an implant, so I went ahead with the reconstruction.”* Some patients also worried that radiotherapy could compromise the aesthetic result. *P1 stated, “I kept wondering whether radiotherapy would affect the implant—make it hard or look worse. If the result deteriorates, would my reconstruction have been pointless?” P6 similarly asked, “I asked the doctor whether radiotherapy would damage the implant. The doctor said it was not certain and depended on individual circumstances. That answer made me even more anxious—would my reconstruction be pointless?”* These accounts indicate that patients often struggle to anticipate the long-term effects of radiotherapy on implants, resulting in a pronounced sense of uncertainty regarding reconstructive outcomes.

##### Uncertainty about the risk of future revision surgery

Patients also reported difficulty in anticipating whether additional surgeries might be needed to maintain or correct the reconstructed breast, creating uncertainty about future treatment demands. Some had been informed preoperatively that implant-related complications could necessitate reoperation, which influenced their decision-making. *P14 expressed, “I worry that if something goes wrong with the implant, I'll have to undergo surgery again. The thought of another operation scares me.”* Others, reflecting postoperatively, reported that they had not fully realized that achieving an optimal outcome might require multiple procedures, which led to psychological distress when faced with potential revision surgery. *P15 said, “I saw cases where patients needed another surgery because of asymmetry after losing or gaining weight, so I started worrying if that would happen to me. Just thinking about a second surgery makes me uneasy.”* These findings suggest that patients' inability to anticipate the possibility of additional surgeries constitutes a unique decision-phase challenge in implant-based breast reconstruction.

#### Theme 2. Conflicting demands of functional recovery and implant shaping

##### Concerns about implant displacement limiting physical activity

In the early postoperative period, patients who underwent axillary surgery faced the dual challenge of promoting upper-limb functional recovery while protecting the implant and surgical site. Striking this balance was a key factor influencing rehabilitation experiences. Participants recognized the importance of gradual arm and shoulder exercises for recovery, yet fear of displacing the implant led some to limit or avoid movements. On one hand, patients wanted to prevent stiffness and maintain mobility; on the other hand, they were concerned that overexertion could compromise implant positioning or aesthetic results. *P6 remarked, “I really wanted to do some arm exercises, but I was afraid that moving too much might shift the implant. I wasn't sure which movements were safe.” P8 similarly said, “I wanted to start moving my arm early; otherwise, it would get stiff. But I was worried about affecting the implant, so I didn't dare try many exercises.” P18 described, “I do wall-climbing exercises every day, but every time I reach chest level, I dare not raise my arm any higher. I keep thinking whether the implant might shift upward with my arm.”* These experiences highlight the tension between functional recovery and implant protection, which significantly shapes patients' rehabilitation behaviors.

##### Discomfort related to wearing shaping garments

Some patients reported difficulties related to wearing shaping garments during the postoperative period. Although participants understood that shaping garments were necessary for maintaining implant position and supporting reconstruction outcomes, prolonged use often caused physical discomfort and inconvenience. Some patients described increased pain or pressure while wearing the garments, while others experienced heat and discomfort that made long-term adherence difficult. Uncertainty regarding the recommended duration of garment use further contributed to inconsistent practices. *P8 described, “After the surgery it was very painful. The doctor said I had to wear the bra, but wearing it made the pain worse. Still, I couldn't stop wearing it.” P10 added, “I don't remember how long the doctor said to wear it. I wore the shaping garment for three months but couldn't see any effect. Later it was too hot, so I stopped wearing it.” P3 shared, “After wearing this shaping garment, I felt constant pressure on my chest. I asked other patients, and they said everyone experiences this and I just had to bear it.”* These experiences suggest that discomfort associated with shaping garments may influence patients' adherence to postoperative shaping recommendations.

##### Limited awareness of early implant-related complications

During postoperative recovery, some patients lacked clear knowledge of early implant- or expander-related warning signs and had difficulty distinguishing abnormal changes from expected healing. Limited discharge education further constrained their ability to monitor complications after leaving hospital. *As P7 reported, “At first, the skin around the reconstruction was a little red. I thought it was normal healing inflammation, so I didn't pay much attention. Later, I learned it was an expander-related infection that could have required removal.” P12 shared, “When I was discharged, the nurse only told me to watch for wound infection. No one ever mentioned that the implant could become hard or deformed. I only learned about this later from searching online.”* These accounts suggest that insufficient early, implant-specific education during the postoperative and discharge period can delay recognition of potentially serious problems.

#### Theme 3. Conflicting care guidance during radiotherapy

##### Conflicting recommendations on shaping garment use during radiotherapy

During adjuvant treatment, patients often faced confusion due to differing care recommendations from different health care professionals. In the early postoperative period, continuous use of a compression bra was commonly advised to maintain implant position and contour. However, during radiotherapy, patients were encouraged to wear loose clothing or reduce pressure on the treated area. The discrepancy between these recommendations created uncertainty in daily self-management. *P9 recounted, “The doctor told me to wear the compression bra for three months, but now the radiotherapy doctor says I should wear loose clothes. I don't know what to do or whether others have experienced this.” P16 also shared, “During radiotherapy, the doctor advised not wearing a bra, but the nurse had told me to keep wearing the shaping bra, otherwise the implant might move. I didn't know whose advice to follow.”* These experiences suggest that inconsistent guidance during adjuvant therapy complicates patients' self-care and represents a key treatment-phase challenge in implant-based breast reconstruction.

##### Cross-phase challenge: difficulty recognizing delayed implant-related complications

Not all complication-related challenges were confined to a single phase. Participants also described difficulties recognizing delayed implant-related complications. Unlike early postoperative concerns such as wound infection or redness, some implant-related complications may become apparent during or after adjuvant treatment and continue to be relevant during long-term follow-up. For example, *P15 said, “I couldn't tell whether the hardness in my implant was related to the treatment. Later, the doctor explained that it was capsular contracture—a term I had never heard before.”* This experience indicates a cross-phase gap in education about delayed implant-related complications that may become apparent during or after adjuvant treatment and continue to be relevant during long-term follow-up.

#### Theme 4. Psychological burden of living with a permanent implant

During the adaptation phase, patients experienced ongoing psychological burden related to living with a permanent implant. Two distinct but related forms of burden were identified: a persistent sense of foreignness, and anxiety triggered by informal information. These psychological challenges often extended well beyond physical recovery and were not automatically resolved once treatment was complete.

##### Persistent sense of foreignness

Some patients continued to experience difficulty integrating the reconstructed breast into their sense of self, even several years after surgery. Although the implant restored body contour, it was often perceived as separate from the natural body. Participants described persistent awareness of the implant's presence, differences in texture and sensation, and feelings of detachment when interacting with their reconstructed breast. *P4 explained, “I tried to accept it and told myself that at least it preserved my appearance. But every time I looked in the mirror, I still felt that side had been ‘attached’ rather than grown as part of me.” Likewise, P11 shared, “My husband accidentally touched that side and asked why it felt so cold. I said it was fine, but inside I felt blocked. What he touched was not the real me.”* These accounts suggest that psychological adaptation to implant reconstruction may extend well beyond physical recovery, with some women continuing to experience a sense of foreignness years after surgery.

##### Anxiety triggered by informal information

Even after completing treatment, some patients remained concerned about the long-term consequences of living with an implant. Information obtained from patient groups or online platforms often triggered uncertainty regarding implant safety and future health outcomes. Although some participants sought clarification from health care professionals, concerns frequently persisted. *P4 explained, “Someone in the patient group shared an article suggesting that implants might be associated with lymphedema. My doctor told me there was no clear evidence, but the statement stayed in my mind.” Likewise, P15 stated, “A patient messaged me saying that someone she knew had an implant removed because of infection and added, ‘You should be careful too.’ That single comment made me anxious for two months.” P2 shared, “I saw a post online saying that implants need to be replaced every ten years. I'm only 47. Thinking about having another surgery in the future makes me very worried.”* These findings indicate that concerns about long-term implant outcomes may persist long after treatment completion and can be amplified by unverified information when ongoing professional support is limited.

## Discussion

### Decision phase: strengthening preoperative information to support multidisciplinary shared decision-making

The findings show that many patients feel uncertain about the long-term effects of implant-based reconstruction. A common concern is how radiotherapy may affect the implant and whether future surgery will be needed. This suggests that gaps remain in reconstruction-specific preoperative counseling. Although routine consultations are provided, many patients still feel unsure about long-term implant management and treatment compatibility. Current education often focuses on the procedure itself, but pays less attention to long-term issues.^18^ Previous qualitative studies have reported similar findings. Women considering breast reconstruction often find it hard to understand treatment trade-offs and long-term expectations when structured decision support is limited.^19^ These uncertainties may be related to how information is provided in clinical practice. Different disciplines often give information separately. Surgeons, oncologists, and nurses may focus on their own areas. This can lead to differences in what patients are told. Patients then need to put this information together on their own. The same difficulty has been described in women's decisions about the type and timing of breast reconstruction, when information from different clinicians is limited and poorly coordinated.^20^ These findings suggest that the issue is not only a lack of information, but also a lack of coordination. Preoperative preparation should go beyond routine surgical consent. More structured and coordinated counseling may help address these challenges. These findings suggest that nurses could provide clear explanations about radiotherapy effects, possible implant problems, and long-term management, and check patient understanding during follow-up. In addition, nurses can help make sure that information is consistent across disciplines and support shared decision-making.^21^, ^22^ Future studies should examine structured preoperative counseling models for implant-based reconstruction. These studies can explore their effects on decision confidence and patient preparedness.^23^, ^24^

### Recovery phase: strengthening coordinated guidance for rehabilitation and implant protection

The findings reveal that during the recovery phase, patients face the critical challenge of balancing necessary arm mobility for functional recovery with the need to protect the newly placed implant and maintain its shape. During the early postoperative period, patients received professional care in hospital, whereas difficulties became more apparent after discharge when they began to manage rehabilitation and shaping care more independently. Patients may also feel unsure about how to interpret postoperative sensations such as pain, tightness, and pressure. This uncertainty may lead to overly cautious behavior or inconsistent adherence to shaping recommendations.^25^ A prospective study of implant-based reconstruction found an early drop in arm strength and patient-reported function, with most patients later regaining at least 80% of their preoperative strength.^26^ Patients’ uncertainty may be related to how rehabilitation guidance is provided. Current guidance often focuses on general surgical recovery. It pays less attention to the specific needs of implant-based reconstruction. Exercise advice and shaping management are often given separately. Patients need to combine this information in daily life, which can be difficult.^27^ This shows a gap between rehabilitation guidance and reconstruction care. In implant-based reconstruction, arm movement and implant shaping need to progress at the same time. Without clear guidance, patients may either limit activity too much or fail to follow shaping recommendations. Clearer and reconstruction-specific guidance may help reduce this problem.^28^ This includes simple instructions on safe activity levels and explanations of common shaping-related sensations.^29^ These findings suggest that nurses could support patients by giving clear and consistent advice on both exercise and shaping, and by explaining early implant-related warning signs (such as progressive redness, warmth, or unexpected changes) at discharge and during early follow-up. Checking how patients perform these tasks and clarifying misunderstandings in a timely way may help reduce avoidable delays in seeking care. Future studies should examine rehabilitation approaches tailored to different reconstruction techniques. These studies can explore their effects on recovery and adherence.^30^

### Adjuvant treatment phase: reconciling conflicting care recommendations

The findings suggest that patients' confusion during adjuvant treatment reflects challenges in aligning guidance from surgical, oncology, and nursing teams. Rather than reflecting individual misunderstanding, the difficulty in balancing implant protection with treatment requirements points to a lack of structured coordination across disciplines.^31^ Previous qualitative studies on breast reconstruction and other complex surgical experiences similarly note that inconsistent recommendations can increase patient anxiety and hinder adherence to care.^32^ In the present study, this issue was particularly evident during radiotherapy, when patients received differing recommendations regarding shaping garment use. These findings highlight the need for nursing interventions that support consistent and comprehensible guidance across disciplines during adjuvant treatment. Challenges in recognizing implant-related complications represent an additional concern that may extend across different phases of the reconstruction journey. Patients' difficulty distinguishing normal recovery from early warning signs, particularly infection, suggests that education may be insufficiently proactive and not adequately adapted to different reconstruction or treatment scenarios.^33^, ^34^ Other studies have shown that delayed introduction of key concepts and unclear explanations can increase psychological burden and delay timely care-seeking.^35^ Some implant-related complications, such as capsular contracture, may become apparent during or after adjuvant treatment and remain relevant during long-term follow-up. Therefore, complication education should not be confined to a single treatment phase. Nurses can play a central role in reinforcing early recognition of complications and providing consistent guidance across the reconstruction journey.^36^ Future programs should integrate phase-sensitive education and real-time support tailored to patients’ reconstruction types and treatment plans, promoting both self-efficacy and safe rehabilitation.^36^, ^37^

### Long-term adaptation phase: strengthening long-term psychosocial support

The persistent psychological burden observed during the adaptation phase suggests that body image acceptance and implant-related anxiety are not resolved automatically once surgery and recovery are complete. This enduring challenge appears linked to the temporal gap between physical reconstruction and psychological adjustment, as well as insufficient continuity in supportive care.^38^, ^39^ Patients often manage concerns related to implant foreignness and conflicting informal information with limited structured professional guidance, which reflects a broader issue: psychosocial concerns may not be sufficiently integrated into the long-term reconstruction pathway. Qualitative studies on breast reconstruction and other chronic conditions support this interpretation, showing that psychological adjustment evolves over months or even years and is highly sensitive to the availability and timing of tailored support.^34^, ^40^ The reliance on informal sources of information further highlights gaps in multidisciplinary communication and follow-up, as patients may lack access to consistent, expert guidance on expected sensory changes, body image adaptation, and realistic long-term outcomes. From a nursing perspective, these findings indicate that one-time postoperative counseling is insufficient. Sustained, stage-specific psychosocial interventions—such as structured follow-up consultations, peer support programs moderated by nurses, and timely clarification of implant-related uncertainties—could mitigate anxiety and enhance body image acceptance.^41^, ^42^ Future research should examine models that integrate psychosocial support throughout the reconstruction trajectory, evaluating their impact on psychological adaptation, adherence to self-care, and quality of life.^43^ These findings also suggest that difficulty recognizing delayed implant-related complications should not be framed as an adjuvant-phase problem alone. Capsular contracture and similar changes may become salient during or after adjuvant therapy and continue to generate uncertainty in long-term follow-up. Sustained, cross-phase education and access to reliable professional clarification may therefore be needed beyond any single treatment stage.

## Implications for nursing practice and research

Based on these challenges, stage-specific nursing support may be needed throughout the implant-based breast reconstruction journey. During the preoperative phase, these findings suggest that nurses could provide individualized decision support and facilitate multidisciplinary communication. During postoperative recovery, structured guidance on rehabilitation exercises and implant shaping management, together with clear education on early implant-related warning signs, may help reduce uncertainty and support safer self-monitoring. During adjuvant treatment, consistent information across disciplines regarding shaping garment use may reduce confusion caused by conflicting recommendations. Because some complication-recognition difficulties span more than one phase, education on delayed problems such as capsular contracture may also be reinforced across adjuvant treatment and long-term follow-up. During long-term adaptation, ongoing psychosocial support and reliable information resources may help patients address persistent concerns and improve adjustment to life with an implant.

## Conclusions

This study provides an in-depth exploration of patient-perceived challenges throughout the implant-based breast reconstruction journey. Four data-derived themes were identified: uncertainty regarding adverse outcomes of implant-based reconstruction, conflicting demands of functional recovery and implant shaping, conflicting care guidance during radiotherapy, and psychological burden of living with a permanent implant. These findings were temporally situated within the reconstruction journey, with some challenges extending across more than one phase. The findings do not directly catalogue supportive care needs; rather, they indicate where nursing support may be most needed. Based on these challenges, nursing care should be continuous, well organized, and responsive across the reconstruction journey. Specifically, nurses may provide clear stage-tailored information, rehabilitation guidance appropriate to the type of reconstruction, stronger multidisciplinary coordination, and ongoing psychological support to help patients cope with long-term recovery. However, this study has limitations. As a single-center qualitative study with a small and relatively homogeneous sample recruited from a tertiary cancer center in Shanghai, the findings may not be transferable to patients from different socioeconomic backgrounds, with lower educational levels, unmarried patients, or those receiving care in other health care settings. Additionally, member checking was limited to two participants and involved review of preliminary findings rather than full interview transcripts. Full transcript validation was not conducted with all participants, which may have limited opportunities to further verify the accuracy of data interpretation. Furthermore, relying on retrospective self-reports may introduce recall or social desirability biases. While Timing Theory usefully informed the temporal framing of data collection and the organization of findings, other theoretical perspectives might offer different insights. Future multicenter and longitudinal studies are needed to further inform the development of stage-specific nursing support.

## CRediT authorship contribution statement

**Xin Zhang**: Investigation, Data curation, Writing – original draft. **Jiaqin Guan**: Formal analysis, Data curation. **Jing Li**: Formal analysis, Data curation. **Jiajia Qiu**: Methodology, Supervision.

**Liqin Chen**: Validation, Writing – review & editing. **Zhenqi Lu**: Conceptualization, Supervision, Writing – review & editing. **Xiaoju Zhang**: Conceptualization, Project administration, Supervision, Writing – review & editing. All authors have read and approved the final manuscript.

## Ethics statement

The study was approved by the Ethics Committee of Shanghai Cancer Center, Fudan University (Approval No. 2509-Exp279). It followed the Declaration of Helsinki. Before the interviews, all participants provided written informed consent. They were informed that participation was voluntary and that they could stop at any time without affecting their treatment or care. Their privacy was protected. All audio recordings and transcripts were stored securely and used only for research purposes.

## Data availability

The data that support the findings of this study are available from the corresponding author upon reasonable request. The data are not publicly available due to privacy and ethical restrictions, as they contain information that could compromise the privacy of research participants.

## Declaration of generative AI and AI-assisted technologies in the writing process

ChatGPT was used for English translation of the manuscript. The authors reviewed and corrected the translated text. All scientific content, study design, data collection, qualitative analysis, interpretation of findings, and conclusions are the work of the authors. The authors take full responsibility for the accuracy, originality, and integrity of the manuscript.

## Funding

This study was supported by the Fudan University School of Nursing Fosun Fund (Grant No. FNF202545). The funders had no role in the study design; data collection, analysis, or interpretation; preparation of the manuscript; or the decision to submit the article for publication.

## Declaration of competing interest

The authors declare no conflict of interest. The corresponding author, Prof. Zhenqi Lu, is an editorial board member of *Asia–Pacific Journal of Oncology Nursing*. The article was subject to the journal's standard procedures, with peer review handled independently of Prof. Lu and the research group.

## References

[1] Sung H., Filho A.M., Laversanne M. (2026). Global cancer statistics 2024: GLOBOCAN estimates of incidence and mortality worldwide for 34 cancers in 186 countries. CA Cancer J Clin.

[2] Zheng R.S., Chen R., Han B.F. (2024). Cancer incidence and mortality in China, 2022. Chin J Oncol.

[3] Xiu B.Q., Guo R., Yang B.L. (2019). Current trends of breast reconstruction after mastectomy in China: a cross-sectional study. Chin J Oncol.

[4] Jonczyk M.M., Jean J., Graham R. (2019). Surgical trends in breast cancer: a rise in novel operative treatment options over a 12 year analysis. Breast Cancer Res Treat.

[5] Yang B., Ren G., Song E. (2020). Current status and factors influencing surgical options for breast cancer in China: a nationwide cross-sectional survey of 110 hospitals. Oncologist.

[6] Kooijman M.M.L., Woerdeman L.A.E., van Duijnhoven F.H. (2025). Short-term complications of immediate implant-based breast reconstruction: risk factor analyses and a novel cumulative risk algorithm. J Plast Reconstr Aesthet Surg.

[7] Santanelli Di Pompeo F., Barone M., Salzillo R. (2022). Predictive factors of satisfaction following breast reconstruction: do they influence patients?. Aesthetic Plast Surg.

[8] Carr T.L., Groot G., Cochran D. (2019). Exploring women's support needs after breast reconstruction surgery: a qualitative study. Cancer Nurs.

[9] Safran T., Al-Halabi B., Viezel-Mathieu A. (2021). Direct-to-Implant prepectoral breast reconstruction: patient-reported outcomes. Plast Reconstr Surg.

[10] Yun J.Y., Jeon D.N., Jeon B.J. (2024). Factors influencing the decision-making process in breast reconstruction from the perspective of reconstructive surgeons: a qualitative study involving Korean plastic surgeons. J Plast Reconstr Aesthet Surg.

[11] Boateng J., Lee C.N., Foraker R.E. (2021). Implementing an electronic clinical decision support tool into routine care: a qualitative study of stakeholders' perceptions of a post-mastectomy breast reconstruction tool. MDM Policy Pract.

[12] Li X., Meng M., Zhao J. (2021). Shared decision-making in breast reconstruction for breast cancer patients: a scoping review. Patient Prefer Adherence.

[13] Cameron J.I., Gignac M.A.M. (2008). “timing it right”: a conceptual framework for addressing the support needs of family caregivers to stroke survivors from the hospital to the home. Patient Educ Couns.

[14] Sandelowski M. (2000). Whatever happened to qualitative description?. Res Nurs Health.

[15] Tong A., Sainsbury P., Craig J. (2007). Consolidated criteria for reporting qualitative research (COREQ): a 32-item checklist for interviews and focus groups. Int J Qual Health Care.

[16] Kallio H., Pietilä A.M., Johnson M. (2016). Systematic methodological review: developing a framework for a qualitative semi-structured interview guide. J Adv Nurs.

[17] Braun V., Clarke V. (2006). Using thematic analysis in psychology. Qual Res Psychol.

[18] Eijsink E.R., Harmeling J.X., Mureau M.A.M. (2023). Decision making in breast implant selection for breast reconstruction: a mixed-method study among plastic surgeons. JPRAS Open.

[19] Li X., Meng M., Yin Y. (2025). Barriers and facilitators for shared decision making in breast reconstruction among stakeholders in the Chinese context: a qualitative study. MDM Policy Pract.

[20] Giunta S., Laidsaar-Powell R., Huang L. (2021). Considering the type and timing of breast reconstruction after mastectomy: qualitative insights into women's decision-making. Eur J Oncol Nurs.

[21] Baithma Jothi D., See M.H., Ng J.H. (2025). Development of a patient decision aid (PDA) for patients undergoing breast reconstructive surgery: towards shared decision-making. Support Care Cancer.

[22] Graziano F.D., White D.R., Plotsker E.L. (2025). Enhancing patient decision-making in breast reconstruction: a systematic review of decision aid efficacy. J Surg Oncol.

[23] Chen L., Lu J., Chen B. (2024). Effect of shared decision-making in patients with breast cancer undergoing breast reconstruction surgery: a systematic review and meta-analysis. Asia Pac J Oncol Nurs.

[24] Yang S., Yu L., Zhang C. (2023). Effects of decision aids on breast reconstruction: a systematic review and meta-analysis of randomised controlled trials. J Clin Nurs.

[25] Higgins M.J., Kale N., Homsy C. (2021). Patient perspective on post-breast reconstruction exercise and physical therapy. JPRAS Open.

[26] Patel A.U., Day S.J., Pencek M. (2020). Functional return after implant-based breast reconstruction: a prospective study of objective and patient-reported outcomes. J Plast Reconstr Aesthet Surg.

[27] Alabaster K., Homsy C., Puyana S. (2021). Exercise after breast reconstruction surgery: evaluating current trends and practices of U.S. plastic surgeons. Plast Reconstr Surg Glob Open.

[28] Campos N.C., Pinto e Silva M.P., Sarmento B.V. (2025). Clinical outcomes of a rehabilitation program following immediate breast reconstruction: a retrospective cohort study. J Manip Physiol Ther.

[29] de Almeida Rizzi S.K.L., Haddad C.A.S., Giron P.S. (2020). Early free range-of-motion upper limb exercises after mastectomy and immediate implant-based reconstruction are safe and beneficial: a randomized trial. Ann Surg Oncol.

[30] Feng Y., Zhang D., Liang F. (2025). [perioperative management of direct-to-implant-based breast reconstruction in breast cancer patients and West China Hospital experiences]. Zhongguo Xiu Fu Chong Jian Wai Ke Za Zhi.

[31] Nava M.B., Benson J.R., Audretsch W. (2019). International multidisciplinary expert panel consensus on breast reconstruction and radiotherapy. Br J Surg.

[32] Fletcher R., Brough G., Macmillan D. (2025). Exploring the informational needs and use of information by patients undergoing risk-reducing breast surgery: a qualitative study. Int J Health Promot Educ.

[33] Mahoney B., Walklet E., Bradley E. (2020). Experiences of implant loss after immediate implant-based breast reconstruction: qualitative study. BJS Open.

[34] Carr T.L., Groot G., Cochran D. (2019). Patient information needs and breast reconstruction after mastectomy: a qualitative meta-synthesis. Cancer Nurs.

[35] Tang S., Anderson N.E., Faasse K. (2022). A qualitative study on the experiences of women with breast implant illness. Aesthet Surg J.

[36] Zhong T., Quong W.L., Cheng T. (2021). Preconsultation educational group intervention can address the knowledge gap in postmastectomy breast reconstruction. Ann Plast Surg.

[37] Webb C., Sharma V., Temple-Oberle C. (2018). Delivering breast reconstruction information to patients: women report on preferred information delivery styles and options. Plast Surg (Oakv).

[38] Bochtsou V., Effraimidou E.I., Samakouri M. (2025). Objective and subjective factors influencing breast reconstruction decision-making after breast cancer surgery: a systematic review. Healthcare (Basel).

[39] Shammas R.L., Fish L.J., Sergesketter A.R. (2022). Dissatisfaction after post-mastectomy breast reconstruction: a mixed-methods study. Ann Surg Oncol.

[40] Zhu L., Miao Q., Li J. (2025). The psychological adaptation journey among Chinese adolescents with inflammatory bowel disease: a qualitative study. Chronic Illn.

[41] Rousset-Jablonski C., Lortal B., Lantheaume S. (2024). French national survey on breast cancer care: caregiver and patient views. Breast Cancer.

[42] Hong W., She Z., Liu X. (2025). A comparative study of quality of life and psychosocial adaptability following modified radical mastectomy and breast reconstruction. Sci Rep.

[43] Bochtsou V., Effraimidou E.I., Samakouri M. (2025). Pre-mastectomy breast reconstruction intentions in women with breast cancer: psychosocial and personality predictors informing mental health promotion. Healthcare (Basel).

